# Speech-language therapy in Kenya: An overview of practitioners, practices and professional needs

**DOI:** 10.4102/ajod.v15i0.1859

**Published:** 2026-05-29

**Authors:** Jinagna Shah, Esedra Krüger, Marien A. Graham, Bhavani S. Pillay

**Affiliations:** 1 Department of Speech-Language Pathology and Audiology, Faculty of Humanities, University of Pretoria, Pretoria, South Africa; 2 Department of Mathematics Education, College of Education, University of South Africa, Pretoria, South Africa

**Keywords:** speech-language therapists, speech-language therapy, practitioners, Kenya, sub-Saharan Africa, practices, needs, survey

## Abstract

**Background:**

Speech-language therapy in Kenya is an emerging profession, yet its workforce practices are not well-understood, and the needs of professionals have yet to be determined. While similar gaps have been reported in sub-Saharan Africa, the Kenyan context remains unknown.

**Objectives:**

To describe current characteristics, practices and needs of speech-language therapists (SLTs) rendering services in Kenya.

**Method:**

An electronic survey was completed by 46 SLTs across Kenya. Descriptive and inferential statistics were used, and open-ended questions were analysed qualitatively.

**Results:**

Most Kenyan SLTs in this sample (78.4%) were located in the urban counties, multilingual and worked across multiple settings. Participants had varied qualifications and backgrounds. The sample included SLTs with overlapping caseloads of client ages – 67.0% served paediatric clients and 53.0% served adults, with many participants reporting that they worked across both groups. Challenges included limited resources (69.6%), high caseloads (52.0%) and a limited workforce. Participants expressed the need for peer support and improved guidelines for practice.

**Conclusion:**

Despite the notable growth of the profession in Kenya, persistent challenges remain within the SLT workforce and structure of service provision. Addressing these requires strengthened collaboration, improved regulation and guidelines and targeted capacity-building to support equitable access to services.

**Contribution:**

This study aimed to outline what the speech-language therapy profession in Kenya looks like, to contribute towards advocating for the profession and planning that can ultimately enhance the impact of SLTs in Kenya.

## Introduction

As members of the allied-health professional community, speech-language therapists (SLTs) support adults and children who face communication and swallowing difficulties in various settings. Currently, in sub-Saharan African (SSA) countries, and Kenya in particular, there is a scarcity of information regarding the structure of the SLT profession and the practices of SLTs (Alighieri et al. 2023; Wylie et al. 2016). To fully understand this newly emerging profession of speech-language therapy in Kenya, it is crucial to understand the characteristics of Kenyan SLTs and their roles when providing rehabilitative services (Wylie et al. 2016). The population of Kenya is rapidly growing, with 53 million people reported in 2022 (The World Bank 2024). An estimated 2.2% of the Kenyan population (aged 5 years and older) were reported to have some form of a disability (Kenya National Bureau of Statistics [KNBS] 2023), and 12% of this group had a communication impairment (KNBS 2023). Other separate studies have indicated that approximately 30% – 50% of their sample groups of children in East Africa have a disability (Hartley et al. 2009; Mung’ala-Odera et al. 2006), but due to the limited sample size of these studies, these figures cannot be generalised to the Kenyan population. However, the above studies all focused on communication disorders, and there is currently no consolidated data available on swallowing disorders in Kenya (Jayes et al. 2024). Given the challenges of screening for communication and swallowing disorders in a low-resource context of the developing world, the true prevalence of these conditions is likely higher than reported (Wylie et al. 2016).

The suggested high prevalence of individuals with communication and swallowing disorders would indicate the need for appropriate and widespread rehabilitation services, including speech-language therapy (Alighieri et al. 2023; Staley et al. 2019). There continues to be insufficient information on the number of SLTs in Kenya. Fagan and Jacobs (2009) led one of the few workforce studies conducted in the SSA region and reported three SLTs, equating to 0.009 SLTs per 100 000 people in Kenya. A follow-up study showed an increase to six SLTs, equating to 0.034 per 100 000 SLTs people (Mulwafu et al. 2017).

The Kenya Health Professions Oversight Authority (KHPOA) is the mandated regulatory body for SLTs in Kenya; however, a publicly accessible record of the number of SLTs is not currently available (KHPOA 2024). At the time of writing this article, the Association of Speech-Language Therapists Kenya (ASLTK), an informal professional group, reported 28 SLTs currently qualified and practising in the country (ASLTK 2024), which corresponds to a ratio of 0.05 SLTs per 100 000 people for a population of approximately 53 million (The World Bank 2024). Comparing this to another SSA region, Pillay et al. (2020) reported that there are 2643 speech-language therapy providing professionals (SLTs and dual-qualified SLT-Audiologists) in South Africa, representing 4.6 SLTs per 100 000 people and a closer professional-to-patient ratio.

Alighieri et al. (2023) researched the paediatric SLT population in East Africa and identified a dearth of services. Fagan and Jacobs (2009) highlighted the shortage of clinical services and expertise in Africa (18 countries, including Kenya) and the need for developing a collaborative global forum, driven by African members (Fagan & Jacobs 2009; Mulwafu et al. 2017). Such recommendations have driven the formation of regional groups like the ASLTK, implementation of these delivery models remains limited by localised barriers. Current research from the SSA region highlights the complexity of the profession, with shortages and migration of SLT professionals, limited local training opportunities and barriers related to language, culture and service delivery models (Alighieri et al. 2023; Staley et al. 2019; Wylie et al. 2016, 2018).

The profession in Kenya started with a handful of expatriate volunteer SLTs in the 1970s and has evolved from isolated practice to a more formal discipline over the decades (Staley & Shah 2023). Local training programmes are now established at a Diploma and Master’s level. The field is currently transitioning towards standardised national regulation under the KHPOA to address the significant gap in service provision. The profession continues to develop, with limited local training programmes and a small workforce. Similar to neighbouring countries in the SSA region and the global South, services have historically been localised within urban regions and there has been limited integration into public systems (Wylie et al. 2016, 2018). Local policies related to disabilities have emphasised inclusion and access to rehabilitation, yet the translation to speech-language therapy specifically remains limited (Sommer et al. 2023).

Other challenges include a lack of standardised practice guidelines and variability in professional training backgrounds (Wylie et al. 2018). Associations and support groups have been formed in East Africa to help guide the profession with assistance from globally practising colleagues (McLeod & Marshall 2023; Wylie et al. 2016). The ASLTK is a professional group of SLTs in Kenya that was created in 2009 to support the practising members and students, though membership is not mandatory (ASLTK 2024). The KHPOA is the mandating body that was formed after the enactment of the June 2017, *Health Act No. 21* to create an ‘effective oversight of the regulation of health standards and provision of health services’ (KHPOA 2024). However, no formal practising guidelines are currently defined. Outlining how SLTs operate within these constraints is crucial to understanding service provision in Kenya.

While studies have identified rehabilitation in the SSA region – specifically the current work profiles while highlighting the scarcity of personnel, resources and barriers to existing services (Alighieri et al. 2023; Wylie et al. 2016) – there remains limited information regarding the Kenyan SLT workforce. This study aims to build on existing evidence from the region by providing a Kenyan-specific overview of current practice, positioning local experiences. To fill these knowledge gaps, this study seeks to provide an updated oversight of SLTs by investigating the following research question: *What are the current characteristics, practices and professional needs of Kenyan speech-language therapists?*

## Research methods and design

### Study design

A predominantly descriptive survey design was used in this study. Given the absence of work addressing this specific topic, a descriptive method was chosen to allow for a more comprehensive understanding of the research question (Hafsa 2019; Swatzell & Jennings 2007). The questionnaire obtained quantitative data using closed-ended questions, supplemented by a few free-text responses and one open-ended question, rendering qualitative data, aimed at providing a comprehensive understanding of the subject matter (Hafsa 2019).

### Setting

Data were collected via an electronic questionnaire circulated using Qualtrics XM. The online format was selected to allow for a larger reach across professionals in the geographical region. All individuals providing communication and swallowing rehabilitation services were invited to participate.

### Inclusion and exclusion criteria

Participants were required to have a university degree (Bachelor’s or Master’s) in speech-language therapy, or have a university degree (Bachelor’s or Master’s) in a closely related field (education, special education, psychology, rehabilitation sciences), followed by SLT training. This broader criterion was selected to capture the diverse workforce of professionals providing communication and swallowing services due to service gaps. Participants were required to be rendering in-person SLT services, astele-rehabilitation was not the focus of this study. To ensure up-to-date and accurate information, all participants had to be currently practising and have a minimum of 6 months of experience providing SLT services. Because the questionnaire was in English, all participants also had to be proficient in English and have internet access to complete the questionnaire online.

### Material

The questionnaire was self-developed, drawing from previously published literature (Wylie et al. 2016, 2018) and was modified to capture information from SLTs who only practice in Kenya with appropriate justifications (Online Appendix 1). Permission to use and adapt the questionnaire was requested and obtained from the authors. To improve face and content validity, the questionnaire was pre-tested with three SLTs working in a similar region, and participants were asked to provide feedback on the content, wording and flow of the questions, multiple-choice answers where applicable, along with ease of filling the questionnaire and acceptable time taken to fill the survey. Based on their feedback, changes were made to the questionnaire regarding wording and answer formats.

In addition, reporting of the online survey followed the CHERRIES (Checklist for Reporting Results of Internet E-Surveys) (Eysenbach 2004) guidelines to ensure transparency in survey design, administration and reporting. The completed checklist is provided as Online Appendix 2. The questionnaire comprised of 30 closed-ended questions, including yes or no format questions, multiple-choice items, Likert scale ratings and opportunities to add free text. The questionnaire concluded with one open-ended question that explored participants’ views about the current services and support available in the country. The survey took approximately 20 min to complete and was accessible for six weeks on the Qualtrics XM software platform. A copy of the questionnaire is available in Online Appendix 3.

### Sampling

Participants were selected via purposive sampling, from the ASLTK group in Kenya who forwarded the cover letter and electronic link to their official membership list. Additionally, snowball sampling was also adopted through social media platforms (such as WhatsApp and Facebook) and professional networks where SLTs were encouraged to disseminate the questionnaire. This approach was necessary due to the absence of a national registry of SLT providers and the dispersion of professionals across diverse work settings. Although ASLTK reported 28 SLTs, this figure only represents SLTs that are affiliated with this group and does not represent the total number of SLTs in Kenya. Potential participants received an introduction to the survey via email, and remaining distributions were via social media channels.

A total of 67 responses were received, of which 21 responses had to be eliminated for not meeting the inclusion criteria (*n* = 6) and for not completing the survey (*n* = 15). Of the six respondents who did not meet the inclusion criteria, two individuals were not practising currently, one provided service only via tele-therapy, and the remaining three did not have the correct qualifications. A total sample of 46 responses was then analysed and reported on.

All included participants were required to be practising SLTs. Most participants were female, within the age range of 21 years to 40 years. Most participants were based within Nairobi county, the country’s capital. Further demographic details are reported in the *Results* section.

### Data analysis

The quantitative data collected were on Qualtrics XM and analysed using Statistical Package for the Social Sciences (SPSS) version 30.0.0. Because all variables were ordinal and not continuous, the nonparametric Spearman correlation (*rs*) was used to identify statistically significant correlations between variables rather than the parametric Pearson correlation (*r*). For the strength of the relationship, the recommendations by Téllez, García and Corral-Verdugo (2015) were used: *rs* < 0.10 (weak), 0.10 < *rs* < 0.30 (weak-to-moderate), *rs* = 0.30 (moderate), 0.30 < *rs* < 0.50 (moderate-to-strong) and *rs* ≥ 0.50 (strong).

Given the exploratory and descriptive nature of this study, corrections for multiple comparisons, such as Bonferroni, were not applied. These adjustments make the interpretation of results contingent on the number of tests performed, introducing arbitrary variability – two authors examining the same hypothesis could reach different conclusions solely based on how many comparisons they conduct (Perneger 1998). Furthermore, Bonferroni corrections are known to be overly conservative, dramatically increasing the risk of Type II errors and potentially obscuring meaningful associations that warrant further investigation (Barnett et al. 2022). Owing to the investigative focus of this study, all correlation analyses are reported with exact p-values and interpreted cautiously rather than mechanically adjusted.

For the free text responses and one open-ended question, qualitative content analysis was applied using open thematic coding. Collected responses were transcribed into a spreadsheet and the raw data were first familiarised by the first author (Mayring 2021). Since most responses were in long paragraphs, shorter key phrases were categorised manually, and repeating ideas were sorted into themes (Puppis 2019). These were then grouped into broader categories through an iterative process. Given the minor volume of qualitative data, coding was only conducted by a single individual (first author), and inter-coder reliability was not undertaken. Representative quotations were selected to illustrate themes based on their clarity and how strongly they replicated participant experiences. No qualitiative analysis software was used.

### Reliability and validity

To enhance the validity and reliability, the questionnaire was pre-tested before being sent out. This ensured the questionnaire questions were unambiguous, enhancing face validity. Content validity was also captured by these peers during the pre-test, ensuring the questionnaire did meet all aspects aptly (Taherdoost 2016). More details on the pre-test are given in the *Material* section. All participants received identical questionnaires, derived from two previously published studies (Wylie et al. 2016, 2018). Results from the mentioned studies are aligned with the current literature exploring speech-language therapy practices in the region, though no studies have been carried out specifically in Kenya (Ndiema et al. 2024). Since the questionnaire predominantly consisted of closed-ended questions, this increased the validity by aiding the criteria for suitable responses to the questions. Since the questionnaire was online and anonymous, there was no pressure on the participant, reducing researcher bias (Brink, Van der Walt & Van Rensburg 2018). The open-ended questions effectively allowed for a deeper analysis of the findings, making them more robust (Swatzell & Jennings 2007).

### Ethical considerations

Institutional ethical clearance was obtained from the Faculty of Humanities’ Research Ethics Committee at the University of Pretoria (reference number HUM025/1124). Voluntary participation was requested from participants. Participants had the right to discontinue the questionnaire at any point before submitting their responses. No incentives were given to encourage participation. The electronic link was accompanied by a cover letter describing the aim of the study, and an informed consent declaration was obtained before beginning the study. To ensure confidentiality of data collected, identifying features such as names of workplace settings or contact details were not collected in the survey. IP addresses were deleted prior to data analysis.

## Results

### Demographics

Thirty-six (78.4%) of the 46 participants were female. Almost half (*n* = 22, 47.8%) of the participants were between 31 years old and 40 years old, followed by 10 (21.7%) participants between 21 years old and 30 years old (Table 1). Most participants (*n* = 36, 78.3%) were working in Nairobi county and city, Kenya’s capital, with several participants indicating they worked in more than one county (Table 1). Fourteen (31.1%) spoke English as their first language, and all participants reported proficiency in at least one other language. Numerous participants (*n* = 24, 52.2%) indicated they did not typically speak the same language as their clients. Participants listed most of their clients as bilingual and multilingual (Table 1).

**TABLE 1:** Age, location (by county) and languages spoken by speech-language therapists in Kenya (*N* = 46).

Characteristics	*n*	%
**Age (years) (*n* = 46)**		
21–30	10	21.7
31–40	22	47.8
41–50	8	17.4
51–60	6	13.0
**SLTs per county reported (*n* = 56) †**, ‡		
Nairobi	36	78.3
Central	6	13.0
Rift Valley	5	10.9
Western	4	8.7
Nyanza	2	4.3
Coast	2	4.3
Eastern	1	2.2
North Eastern	0	0.0
**Summary of first language of SLTs (*n* = 45) †, §, ¶**		
English	14	30.4
Swahili	6	13.0
Kikuyu	8	17.4
Luhya	3	6.5
Ekegusii	3	6.5
Dholuo	2	4.3
**Summary of other languages SLTs were proficient in †, ¶**		
English	36	-
Swahili	36	-
Kikuyu	8	-
Luhya	4	-
Kamba	2	-

† , Participants could select more than one response;

‡ , Several participants reported working in more than one county;

§ , Some answers were incomplete;

¶ , Full table is available in Appendix 1.

The majority of participants (*n* = 43, 93.5%) reported having formal training in the field of speech-language pathology and/or therapy, and nearly half (*n* = 21, 45.7%) were registered and licensed with the KHPOA. It is to be noted that individual licensing was self-reported though not verified, as a publicly accessible record is not currently available. Most participants held a Master’s degree (*n* = 27, 58.7%) or Bachelor’s degree (*n* = 12, 26.1%), while a few were still awaiting their Master’s certification (*n* = 3, 6.5%). More than half of the participants (*n* = 26, 56.5%) were employed full-time, primarily in schools (*n* = 18, 39.1%) and private practice clinics (*n* = 12, 26.1%), among a variety of other settings like home health and hospitals (Table 2). More than half of participants (*n* = 26, 56.5%) self-identified as working full time. However, many of these participants reported providing services across multiple settings and counties, reflecting a consultancy-based employment rather than single-site positions.

**TABLE 2:** Work settings and prior training for speech-language therapists (*N* = 46).

Workplace settings	*n*	%
**Primary workplace settings of SLTs (*n* = 46)**		
School (in special needs department)	18	39.1
Private practice clinic	12	26.1
Hospitals	9	19.6
Home health	3	6.5
University or college	2	4.3
Community-based work	2	4.3
**Other settings SLTs also worked in†**		
Home health	17	37.0
Private practice clinic	14	30.4
School (in special needs department)	14	30.4
Private hospital	11	23.9
Telehealth	6	13.0
Government hospital	3	6.5
University or college	1	2.2
NGO or Community Centre	1	2.2
Mission hospital	1	2.2
Nursing home	1	2.2
**Prior training in any other field (*n* = 46)**		
Yes	35	76.1
**Participants who had prior training in the following fields**		
Special needs education	9	-
Counselling or Human psychology	4	-
Education	3	-
English literature or Linguistics	2	-
Occupational therapy	2	-
Clinical Medicine	1	-
Nursing	1	-
Paramedics	1	-

† , Participants selected more than one response.

Approximately three-quarters of the participants (*n* = 35, 76.1%) also had prior training in other fields; for example, nine of the 35 (25.7%) had prior training in special needs education. Experience levels varied, with half of the participants having between 1 year and 5 years of experience, and eight participants having between 6 years and 10 years of experience. The remaining participants had over 10 years of experience. Participants worked in various settings, and some also had prior training in other fields (Table 2).

### Speech-language therapists’ practice

**FIGURE 1:**
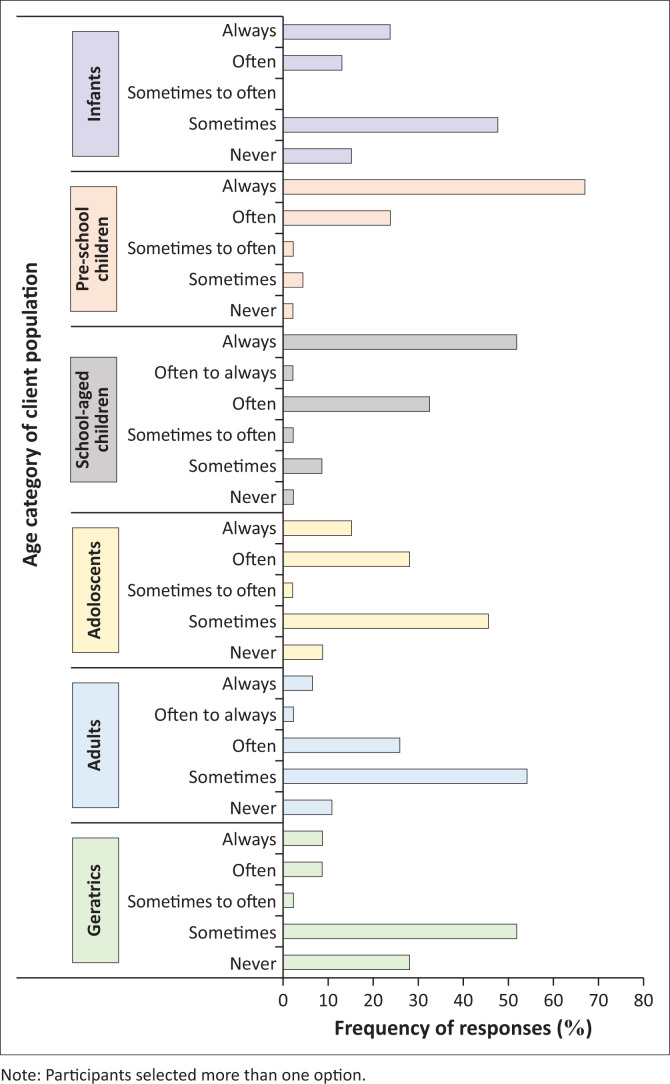
Frequency of speech-language therapists providing services? across various age groups.

Almost half of the participants (*n* = 22, 47.8%) saw up to six clients a day. Approximately one-quarter (*n* = 12, 26.1%) had a caseload of up to three clients per day. Two participants (4.3%) reported seeing 10 to 15 clients or more daily. Figure 1 shows the frequency with which client populations across various ages were seen. For example, more than half (*n* = 31, 67.4%) of the group reported they *always* saw pre-school-aged children. The majority of participants reported working with paediatric clients, with approximately two-thirds (*n* = 31, 67.4%) *always* seeing pre-schoolers and around a half (*n* = 24, 52.2%) *always* working with school-aged children. Inferential statistics via the Spearman test revealed there was no statistically significant correlation between the number of years of experience the therapist had and the age of clients they serve, as the value of *p* was larger than 0.05.

The participants’ caseloads are shown in Table 3. The main paediatric services rendered were to children with autism (*n* = 43, 93.5%), articulation or phonological disorders (*n* = 39, 84.8%), need for early communication intervention (*n* = 37, 80.4%) and fluency disorders (*n* = 31, 67.4%). Service delivery to adults included management of aphasia (*n* = 29, 63.0%), dysarthria (*n* = 20, 43.5%), stroke-related speech and communication difficulties (*n* = 25, 54.3%), fluency disorders (*n* = 24, 52.2%) and voice disorders (*n* = 22, 47.8%). Based on their training, support and professional development, approximately half of the participants (*n* = 23, 52.3%) *felt confident* in taking on the caseloads that they indicated. A third of participants (*n* = 14, 31.8%) felt *very confident*, whereas a smaller group (*n* = 6, 13.6%) felt *somewhat confident*. Two participants (4.3%) did not respond.

**TABLE 3:** Caseloads that speech-language therapist providers are rendering services to (*N* = 46).

Client caseload†	*n*	%
Autism in children	43	93.5
Articulation or phonological disorders in children	39	84.8
Pragmatics or social communication	38	82.6
Early communication intervention in children	37	80.4
Semantics, morphology and syntax	36	78.3
Reading and writing difficulties in children	32	69.6
Fluency disorders in children	32	69.6
Aphasia in adults	29	63.0
Cerebral palsy in children	28	60.9
Stroke in adults	25	54.3
Hearing loss in children	24	52.2
Fluency disorders in adults	24	52.2
Childhood apraxia of speech	22	47.8
Voice or resonance disorders in adults	22	47.8
Cleft lip and/or palate and other craniofacial anomalies in children	20	43.5
Dysarthria in adults	20	43.5
Dysphagia in adults	17	37.0
Dysphagia in infants and children	15	32.6
Apraxia of speech in adults	15	32.6
Cognitive-communication disorders related to degenerative conditions, i.e. dementia in adults	12	26.1
Head and neck cancer in adults	7	15.2
Autism in adults	7	15.2
Cleft lip and/or palate and other craniofacial anomalies in adults	6	13.0
Cerebral palsy in adults	5	10.9
Other (Down syndrome)	1	2.2

† , Participants selected more than one option.

For these ordinal values, the nonparametric Spearman correlation was conducted to identify any correlation between the number of years of experience and the level of confidence the participant had (*rs* = 0.20, *p* = 0.201). There was no statistically significant correlation seen between these variables. The Spearman correlation was also applied to explore any correlation between the level of confidence of the participant and the types of caseloads they served. There was a statistically significant moderate positive correlation between the level of confidence for a dysphagia caseload (*rs* = 0.30, *p* = 0.048) and a moderate-to-strong positive correlation for cerebral palsy (*rs* = 0.35, *p* = 0.019). There was no statistically significant correlation between the level of confidence and any other caseloads (*rs* = 0.20, *p* = 0.200), since the *p*-value was greater than 0.05.

For the number of hours per week, including direct and indirect speech-language therapy services (e.g. planning, administration, report writing, supervision, educating and collaborating with team members), nearly one-third of participants (*n* = 14, 30.4%) reported working between 25 and 40 h weekly, while just over a quarter (*n* = 12, 26.1%) reported working more than 40 h per week. Eleven participants (23.9%) reported working between 15 and 25 h a week, and the remaining (*n* = 9, 19.6%) reported working less than 15 h weekly.

Most participants (*n* = 41, 89.0%) reported working in departments with only one to three SLTs. The remaining five participants (10.9%) indicated there were four to six SLTs at the workplace. The majority of participants (*n* = 30, 65.2%) felt that the number of SLTs in their workplace was insufficient to cover the current caseload demands. Participants cited contributing factors of high caseloads, time constraints and low number of SLTs, as attested to in the following statements:

‘There are more clients needing intensive input than the time available for the few speech therapists.’ (Participant 16, Nairobi county private practice SLT, 11–15 years’ experience)

In some areas, only one SLT in the surrounding geographical region was reported:

‘I am the only available speech therapist in the entire county.’ (Participant 6, full time at a school setting, 1–3 years’ experience)

‘Not at all, I am the only one. Work is overwhelming.’ (Participant 18, multiple counties part time at hospital setting, 11–15 years’ experience)

Conversely, more than a third (*n* = 16, 34.8%) reported having an adequate number of SLTs for their caseloads. The reasons reported for this were a lower caseload and an increased number of providers in the capital city.

Primary work functions for SLTs included assessment (*n* = 41, 89.1%) and treatment (*n* = 44, 95.7%) of clients. (Table 4). Professional collaboration included working with occupational therapists (*n* = 43, 93.5%), teachers (*n* = 33, 71.7%), physiotherapists (*n* = 27, 58.7%), medical practitioners (*n* = 25, 54.3%), audiologists (*n* = 24, 52.2%), psychologists (*n* = 20, 43.5%), dieticians (*n* = 17, 37.0%), nurses (*n* = 14, 30.4%), social workers (*n* = 11, 23.9%) and behavioural technicians (*n* = 1, 2.2%). Over half of the participants (*n* = 25, 54.3%) also rendered group therapy as part of their services, where two participants (4.6%) indicated mainly facilitating groups for child language therapy.

**TABLE 4:** Primary and secondary work functions of speech-language therapist at their workplace (*N* = 46).

Functions of SLTs at work	*n*	%
**Primary functions** †		
Treatment	44	95.7
Assessment	41	89.1
Consultation	31	67.4
Screening	23	50.0
Teaching	18	39.1
Administration	17	37.0
Others	2	4.3
**Secondary functions?**		
Professional collaboration	34	73.9
Case Management	25	54.3
Research	21	45.7
Teaching	18	39.1
Administration	13	28.3
Screening	13	28.3
Others	1	2.2

† , Participants selected more than one option.

### Professional needs

Regarding SLT service provision, 19 (41.3%) participants reported having in-house clinical protocols, such as standard operating protocols, assessment and screening tools. In contrast, 15 participants (32.6%) did not have any workplace guidelines, and the remaining were unsure (*n* = 9, 19.6%). Some participants (*n* = 3, 6.5%) noted that protocols did not apply to their specific setting.

The majority of participants (*n* = 37, 84.0%) agreed that Kenyan SLTs required national clinical guidelines for the assessment and treatment of specific disorders. In the free-text section of this question, multiple themes were identified: the need for standard, evidence-based guidelines, culturally and linguistically appropriate assessment protocols, and improved practice protocols. Barriers to service delivery and the need for affordable continuing professional educational opportunities were also expanded on.

Participants identified the need for standard guidelines for the profession, as well as the need for more specific, evidence-based guidelines outlining specific disorders:

‘SLT[s] need to have the national clinical guidelines of specific disorders to make it possible to specialise in specific areas.’ (Participant 17, school-based SLT, 3–5 years’ experience)

Participants felt that guidelines would be beneficial, especially to guide the newly qualified professionals in the country:

‘Guidelines for the Kenyan population would help provide evidence-based care for our patients. It may be particularly helpful to newly qualified practitioners (NQPs) and those who do not usually work [*in*] that area of practice.’ (Participant 39, part-time school-based SLT, 11–15 years’ experience)

A need for culturally and linguistically appropriate assessment protocols was identified:

‘This would lead to having assessment tools for different client groups, which would align with the Kenyan culture.’ (Participant 31, hospital setting, 3–5 years experience)

Participants also recognised the need for improved standards of practice and for curbing unqualified professionals from performing the SLTs scope:

‘Yes, to have a standard [*guideline*] of work and something to govern people practising as there are many unqualified persons undertaking the job.’ (Participant 7, part-time SLT in private practice and schools, 7–10 years’ experience)

The need for uniformity for service provision amongst SLTs was also identified:

‘We need to have similar ways of handling communication disorders for the benefit of the clients.’ (Participant 14, private practice, 4–5 years’ experience)

The requirement for structure and quality of care also emerged:

‘Yes, to ensure professional standards are operationalised and upheld, thus safeguarding the clients and the profession as it relatively new.’ (Participant 32, private practice, 1–3 years’ experience)

However, three participants were of the opinion that current local guidelines needed refinement rather than adopting ready guidelines from developed countries, due to a varied population:

‘Kenya has a diverse population from which clients come. No [*few*] sets of guidelines [*available*] would represent that diversity and enable good clinical input or practice.’ (Participant 16, part time hospital and private clinic-based SLT, 7–10 years’ experience)

The key barriers to service delivery participants recognised were as follows: lack of resources (*n* = 32, 69.6%), a high caseload (*n* = 24, 52.2%) and travelling across different geographical locations (*n* = 21, 45.7%) as indicated in Table 5. In an open-ended section that probed further, one participant reported that:

‘… most marginalised communities are missing out on most of the services due to the limited number of trained speech therapists in the country and limited resources making it difficult to ensure early intervention.’ (Participant 27, part-time school-based SLT, 1–3 years’ experience)

**TABLE 5:** Barriers to speech-language therapist service delivery (*N* = 46).

Barriers†	*n*	%
Lack of resources	32	69.6
High caseload	24	52.2
Travelling across different settings in different geographical locations	21	45.7
Time pressures	16	34.8
I feel under-skilled in some areas	15	32.6
Lack of training	13	28.3
Varied caseload	12	26.1
Cost burden on clients	5	11.0
Difficulty accessing professional supervision	3	6.6
Services by unqualified professionals	2	4.3
Poor government support	2	4.3
Lack of relevant assessment tools	1	2.2
Poor awareness	1	2.2
Lack of specialist SLTs	1	2.2

† , Participants selected more than one option.

Speech-language therapists reported a lack of an adequateworkforce for the high caseload of clients. An uneven urbandistribution was seen:

‘… given that speech therapist[*s*] are scarcely spread out all over the country with most of them concentrated in Nairobi.’ (Participant 7, part-time SLT in private practice and schools, 7–10 years’ experience)

Access to services was limited for clients due to financial factors:

‘… there is a lack of contextualised materials for local communities, services are only available primarily to individuals with disposable income.’ (Participant 32, private practice, 1–3 years’ experience)

Speech-language therapists also reported:

‘… there are limited resources [*and*] opportunities for growth.’ (Participant 41, private practice, 3–5 years’ experience)

All participants (*n* = 46, 100%) reported regularly attending continued professional development (CPD) and training to improve their skills and knowledge. Participants preferred webinars (*n* = 39, 84.8%), short courses (*n* = 37, 80.4%) and in-person workshops (*n* = 30, 65.2%). Participants also noted self-directed study, specific treatment approaches or technique courses, professional groups and mentorship with colleagues as part of their development. The increased access and opportunities for CPD provided by groups such as the ASLTK were acknowledged by some participants (*n* = 12). However, access to these CPDs was limited by time and funding:

‘Because the structure of working in Kenya is based on a consultancy rather than an employment framework, it is the responsibility of each SLT to take up CPD at their own time and cost. With high caseloads, this can often be a challenge.’ (Participant 43, part-time community-based SLT, over 15 years’ experience)

There was a call for more specialised and apt continuing professional development opportunities:

‘The ASLTK offer CPD but this can sometimes be pitched at a lower level, which is great for educating newly qualified therapists but it is often not to all levels.’ (Participant 7, part-time SLT in private practice and schools, 7–10 years’ experience)

Culturally and linguistically appropriate learning opportunities were also mentioned:

‘Many of the continued education opportunities are great and evidence-based but [are] not contextually applicable to the extent of that knowledge being effective [*in the Kenyan work setting*].’ (Participant 16, part time hospital and private clinic based SLT, 7–10 years’ experience)

Responses to the open-ended question explored views regarding current services and support available in the country: specific needs, continued education opportunities, support from professional bodies, and opportunities to collaborate and learn. Many participants (*n* = 6) recognised the growth of the SLT field in Kenya over the last few years:

‘… the profession, though growing slowly, is making great strides.’ (Participant 1, private practice, over 15 years’ experience)

There has been an improvement in the number of training programmes in the country; however, there is a lack of a well-established, government-supported regulatory body:

‘We need structures put in place to grow the professional body that governs SLTs in Kenya.’ (Participant 22, private practice, 10–15 years’ experience)

‘The support currently offered in these areas is not adequate. The regulatory body is still in its infancy stage and in the process of developing professional guidelines.’ (Participant 43, part-time community-based SLT, over 15 years’ experience)

‘There is support from professional bodies though not sufficient, [*and*] none from government’ (Participant 18, multiple counties part time at hospital setting, 11–15 years’ experience)

‘There are now more training programmes locally and positive [*for*] more SLTs for growing population with needs. However, [*the*] quality [*of the programmes*] needs to be looked into. Mentorship and apprenticeship programmes should be created to support regulated services.’ (Participant 34, school-based SLT, 6–10 years’ experience)

‘More collaborations and support to our local universities will improve the training and quality of our SLTs graduating locally.’ (Participant 3, private practice, 3–5 years’ experience)

There is also need for mentorship:

‘… more support to ensure that the newly qualified SLTs are well prepared to take up their role in therapeutic fields.’ (Participant 13, private practice, over 15 years’ experience)

Additionally, some participants (*n* = 10) reported the need for more peer support, mentorship and interdisciplinary collaboration to combat segregation and improve service quality:

‘Though the field is growing, there’s great need for proper mentorship, collaboration by members and teamwork with allied professionals for an interdisciplinary approach.’ (Participant 10, school-based SLT, 6–10 years’ experience)

‘[*There is*] limited opportunity for collaboration as people [*are*] working often alone and many [*are*] not keen to collaborate on cases.’ (Participant 5, hospital based SLT, 11–15 years’ experience)

‘SLT is mostly private and not salaried. This makes it unnecessarily competitive. Knowledge, quality therapists and resources are gate-kept even though there’s enough work for everyone. No room to develop specialisms as everyone is doing everything and [*there are*] less opportunities to collaborate.’ (Participant 24, hospital-based, 1–3 years’ experience)

This leads to isolation in the profession:

‘SLTs throughout the country might benefit from efforts to collaborate more - especially those who might be more isolated in rural areas.’ (Participant 1, private practice and part-time community-based SLT, over 15 years’ experience)

To further improve support one participant noted:

‘… teamwork with allied professionals for an interdisciplinary approach [*would be beneficial*].’ (Participant 10, school-based SLT, 6–10 years’ experience)

Participants also recognised the increased support from both regional and global groups of SLTs, such as the *African Connections Group* and the *International Association of Communication Sciences and Disorders*.

## Discussion

This study describes the characteristics, practices and needs of 46 Kenyan SLTs. The sample comprised predominantly female participants (78.4%), aligning with gender disparity in the SLT profession globally; for example, the Royal College of Speech and Language Therapists (2020) reports only 2.5% of male SLTs in the United Kingdom. Most of the workforce in this sample is young and has less than 5 years of experience, echoing the recent establishment of Diploma and Master’s training programmes in state universities in Kenya and the broader SSA region (Alighieri et al. 2023; Olszewski et al. 2023). The small number of SLTs with over 10 years of experience showcases the limited pool of experienced mentors for the growing SLT population, which mirrors findings from the East-African context (Marshall & Wickendon 2018). There is an increasing need for SLTs and a high demand that currently cannot be met (Mwende & Abuom 2024; Staley & Shah 2023).

The concentration of most SLTs in Nairobi reflects a higher number of professionals in the capital city and an urban setting, where there are likely more professional opportunities (Machio 2016). This similarity is also seen in other studies within the SSA region (Wylie et al. 2016, 2018). The imbalance of health care workers (Machio 2016) and specifically SLTs, within urban vs rural settings, also suggests there is more peer support and ease of referrals within the capital, whereas those working in rural counties could be potentially secluded (Obura 2014; Staley et al. 2019). However, SLTs describing working in multiple locations and counties suggest some degree of mobility and reflect the recent growth in Kenya’s SLT workforce and evolving service demands, covering regions outside the urban capital. Future research is required on this rural-urban distribution and how this impacts the client populations.

Recognition of SLTs and training programmes has grown over the last few decades (Fagan & Jacobs 2009). Participants repeatedly emphasised the lack of structural and regulatory support for the profession. As local programmes continue to grow, formal frameworks to support NQPs remain limited (Olszewski et al. 2023; Wylie et al. 2016), underlying the need for further policy planning and professional guidance. Qualitative responses pointed to perceived gaps between academic training and clinical practice, with limited mentorship. Regarding research, there is a need for more local guidelines based on evidence-based practice. The need for locally relevant, evidence-based guidelines was frequently raised, aligning with broader calls for professional development across SSA (Nthiga & Nyamasyo 2023; Wylie et al. 2018).

Most SLTs provide services in the private sector (home health, schools and private clinics or hospitals), with only a handful in government hospitals. Government recognition and support for the profession have been emerging over the last decade (Nthiga & Nyamasyo 2023; Sowden et al. 2023; Staley et al. 2021), and government job opportunities are still scarce. Speech-language therapists also work in multiple settings (Wylie et al. 2016), often on a part-time contractual basis, rather than full-time employment, suggesting a need to supplement income and highlighting potential limitations in full-time job opportunities.

Speech-language therapists completed their first degrees in a variety of backgrounds, frequently the educational, medical and allied-health fields (Wylie et al. 2016, 2018). This reflects in the choice of their work setting: a large group of SLTs with teaching backgrounds work as SLTs in schools and serve a paediatric population (Alighieri et al. 2023). A high proportion of SLTs consistently working with pre-school and school-aged children reflects global trends in early intervention (Blackman 2002), with many Kenyan SLTs coming from education and special education backgrounds.

Most participants had a Master’s degree, though information regarding their training location was not collected in this study. Qualitative responses identified the need to protect the profession from unqualified practice. While Kenyan SLTs have historically lacked a national regulatory and legislative framework (Nthiga & Nyamasyo 2023; Staley & Shah 2023), the newly introduced KHPOA is poised to change this and represents a direct implementation of regional recommendations (Fagan & Jacobs 2009). However, the low number of licensed professionals indicates potential barriers, which can be researched further. Ensuring that rehabilitation services, including SLTs, are regulated is crucial to prevent individuals with communication and swallowing disabilities from receiving inappropriate and unstandardised care. While countries like South Africa possess well-established regulatory frameworks, the lack of centralised government licensing remains a challenge in other sub-Saharan countries, including Kenya (Alighieri et al. 2023; Mehmood 2023; Ned et al. 2020). Additional research is needed to understand the factors influencing the uptake of licensure and its impact on standardising professional practices across the country.

While English and Swahili are seen as the official languages of Kenya, there are over 50 indigenous languages (Barasa 2023). The multilingual nature of the Kenyan SLT in this sample is a common characteristic across many African countries (Dwivedi 2014; Wylie et al. 2016, 2018), and yet therapists may not necessarily speak all the same languages as their clients. This linguistic mismatch, for example, the need for translation services, is likely to pose challenges in access, service delivery and shared decision making, hence affecting therapy outcomes (Mophosho 2018; Staley et al. 2021). There is a need for continued investigation into the polyglottic Kenyan scenario and SLT service delivery.

Challenges SLTs face are the lack of culturally and linguistically appropriate tools for service provision, and these findings reinforce the need for culturally and linguistically appropriate assessment and intervention tools reported from the region (Hartley et al. 2009; Mwende & Abuom 2024; Ndung’u & Kinyua 2009; Sowden et al. 2023; Staley et al. 2021; Wylie et al. 2016). Assessment and intervention tools originating from Western settings should not be directly transplanted but rather modified to adapt to and reflect the cultural sensitivity of the population (Carter et al. 2012; Ndung’u & Kinyua 2009; Staley et al. 2019). The same principle applies to guidelines and training opportunities, which is later discussed. There is a significant need for further research in developing these tools, relevant to the diverse Kenyan populations.

Although a high proportion of participants reported collaboration with their multi-disciplinary team, qualitative findings suggest that this collaboration is often client-specific. This highlights an important distinction between interdisciplinary collaboration and broader professional peer support, and mentorship within the profession itself. With most therapists being in the private sector, there is a vast population of clients who cannot access these likely higher-priced, privatised services. Challenges in obtaining these resources would also result in unequal access to services across the country (Eikerling et al. 2025; Mwende & Abuom 2024). This may warrant further research to determine whether this inequality affects certain socio-economic groups requiring communication and swallow rehabilitation.

Speech-language therapists in this sample serve varied caseloads each day, and working hours for SLTs vary from 15 to more than 40 h of direct services weekly. Some SLTs reported high caseloads while others reported smaller ones, but interestingly, many expressed being overwhelmed due to their workload. While this finding may not show all SLTs to have actual high caseloads daily, it may allude to SLTs not coping with the work they deal with daily, which could be explained by development, leadership, support and systems difficulties in work settings. However, this would have to be explored in future research. While 34.8% of participants reported feeling adequately staffed, the current data did not allow for a cross-analysis to determine if these perceptions were linked to specific work settings, such as private vs. public sectors. Consequently, this finding contradicts broader evidence of workforce shortages across Kenya and the region and can be researched further.

No SLTs in this group specialise in any particular client case populations. Constraints related to resources, geography and workforce size may limit opportunities for specialisation, particularly outside urban centres (Mwende & Abuom 2024; Staley et al. 2019). These findings are similar to broader concerns about health services in many low-income countries (Wylie et al. 2018). Contextually relevant research in Kenya and sub-Saharan Africa is required to address these gaps in service provision.

Most Kenyan SLTs in this data set have been trained within the last 5 years and report varied low-to-medium confidence levels in their work. Although years of experience did not significantly correlate with the therapists’ overall confidence, the broad range of client populations managed by clinicians indicates a need for professional supervision and mentorship structures to support quality care across these diverse settings (Jackson, Purdy & Cooper-Thomas 2019; Staley et al. 2019). Although participant experiences, for example, feelings of burnout, were not directly measured in this study, existing studies suggest that high caseloads may contribute to occupational strain and limit access to care (Okech, Chokwe & Mung’ayi 2015). It would be beneficial to conduct further research into local SLTs’ perceptions to better understand these needs.

Speech-language therapists recognise the importance of professional development. Their preference for webinars and short courses points to significant time and financial constraints. They acknowledge the need for different levels of training that can support both NQPs and experienced clinicians looking to specialise (Wylie et al. 2016). The universal reporting of CPD participation by all SLTs in this sample may reflect social desirability bias inherent in self-report surveys rather than actual representation. However, many existing CPD opportunities, while evidence-based, are not suitable for the Kenyan context due to a lack of cultural and linguistic relevance (Wylie et al. 2023). More inquiry is needed to address this gap, and leadership within the SLT community must take this on.

Many SLTs lack clinical protocols at their workplaces, highlighting the need for proper institutional frameworks. Guidelines must be culturally and linguistically appropriate for Kenya’s diverse environment (Carter et al. 2012; Staley et al. 2019). Without standardised practices, therapists can experience reduced job satisfaction (Wylie et al. 2018). Rather than reflecting individual practitioner shortcomings, these findings point to systemic gaps in guideline availability and workforce support. There is a clear demand for structured employment policies in both private and public sectors to support full-time work. Given the reported absence of clinical protocols in many workplaces, the findings regarding national guidelines point to an immediate area for policy development. There is a clear demand for evidence-based guidelines, both for the profession as a whole and for specific disorders, to improve the quality of care provided.

### Limitations

This study used purposive and snowball sampling to recruit participants. This resulted in a disproportionately urban-based group, and the authors acknowledge that selection bias could be present. As such, the findings reflect the experiences of the current sample rather than the entire national workforce, potentially limiting their generalisability to the wider population of Kenyan SLTs (Andrade 2021). It should also be noted that participants with diplomas in speech therapy were not included in the study, as these programmes were not known to the authors during the initial phase of disseminating the survey. The truthfulness of the results is reliant on how accurately the participants self-interpreted and responded to survey questions. However, the study did not independently verify professional qualifications or licensure status, and these factors may contribute to variability in professional status within the sample. Data were self-reported and may be influenced by recall or social desirability bias. Although the inherent limitations of self-administered online surveys remain, adherence to CHERRIES reporting standards enhanced transparency regarding survey administration and response patterns.

The survey instrument was adapted from existing literature but did not undergo formal psychometric validation. Similarly, the qualitative component was based on a limited number of open-ended survey responses, and inter-coder reliability was not calculated, thus qualitative findings should therefore be interpreted as illustrative. Even though the number of SLTs in this study is a fair sample size, a post-hoc power analysis using G*Power software (Faul et al. 2007) indicated that 84 respondents would be required to achieve a power of at least 0.80 for correlation analyses at a significance level of 0.05 to detect at least a medium effect size. This study included only 46 respondents, which means the statistical power was lower than recommended. As such, correlation findings should be interpreted cautiously, particularly where effect sizes were small. Despite these limitations, this study offers an insight into practices and challenges SLTs face in Kenya.

## Conclusion

This study shows the reality of speech-language therapy in Kenya, a rapidly growing profession, but hindered by persistent systemic challenges. This small-scale study described a sample of the workforce and identified the highly variable nature of the Kenyan SLT. Similar to patterns in the SSA region, SLTs were multilingual and urban-centric. Speech-language therapists were trained in a variety of roles and worked in transient settings, serving a wide pool of clients and disorders.

There is a growing local workforce with adequate potential for covering the service gaps via local training programmes. However, the lack of a well-established clinical infrastructure directly affects this service delivery. National attention and strategic planning for the SLT profession is now indicated, and standardisation across clinical practice is essential. Collaborations between government, private networks and academic stakeholders are key in enhancing the standardisation of care across the diverse Kenyan setting.

Effective collaboration must include government-led creation of public-sector SLT positions, standardising both public- and private-sector frameworks and aligning curricula of local programmes with the clinical realities. Furthermore, building SSA networks, such as a regional clinical-mentorship exchange, is vital for sharing culturally responsive assessment tools and providing peer supervision; this is a challenge that local SLT leadership groups are well-positioned to lead. This study provides a foundation for advocating for the profession and informing academic and local policy planning that can ultimately enhance the impact of speech-language therapy in Kenya.

## Appendix 1

**TABLE 1-A1:** Language proficiency among Speech-language therapist (SLT) participants.

Category	*n*	%
**First language of SLT participants (*n* = 45)†**		
English	14	30.4
Swahili	6	13.0
Kikuyu	8	17.4
Luhya	3	6.5
Ekegusii	3	8.7
Dholuo	2	2.2
Kamba	1	2.2
Embu	1	2.2
Kimeru	1	2.2
Kipsigis	1	2.2
Kisii	1	2.2
Luganda	2	4.3
Punjabi	1	2.2
Taita	1	2.2
**Other languages SLTs were proficient in** ‡		
English	36	78.3
Swahili	36	78.3
Kikuyu	8	17.4
Luhya	4	8.7
Kamba	2	4.3
Dholuo	2	4.3
Kimeru	3	6.5
Kalenjin	0	0.0
Maasai	0	0.0
German	1	2.2
Gujarati	2	2.2
Hindi	1	2.2
Kenyan Sign Language	3	6.5
Kisii	1	2.2
Luganda	1	2.2

† , Some responses were missing;

‡ , Participants selected more than one language.
